# Exploring the Effects of Various Capping Agents on Zinc Sulfide Quantum Dot Characteristics and In‐vitro Fate

**DOI:** 10.1002/open.202300094

**Published:** 2023-10-06

**Authors:** Zohre Montaseri, Ali Mohammad Tamaddon, Mohammad Javad Raee, Fakhrossadat Farvadi

**Affiliations:** ^1^ Center for Nanotechnology in Drug Delivery Shiraz University of Medical Sciences Shiraz Iran; ^2^ Department of Pharmaceutical Nanotechnology Shiraz University of Medical Sciences Shiraz Iran

**Keywords:** quantum dot, colloidal stabilization, capping agent, stabilizing agent, nanoparticle fate

## Abstract

The choice of capping agents used during the synthesis process of quantum dots (QDs) can significantly influence their fate and fundamental properties. Hence, choosing an appropriate capping agent is a critical step in both synthesis and biomedical application of QDs. In this research, ZnS QDs were synthesized via chemical precipitation process and three commonly employed capping agents, namely mercaptoethanol (ME), mercaptoacetic acid (MAA), and cysteamine (CA), were used to stabilize the QDs. This study was aimed to examine how these capping agents impact the physicochemical and optical characteristics of ZnS QDs, as well as their interactions with biological systems. The findings revealed that the capping agents had considerable effects on the behavior and properties of ZnS QDs. MAA‐QD exhibited superior crystal lattice, smaller size, and significant quantum yield (QY). In contrast, CA‐QDs demonstrated the lowest QY and the highest tendency for aggregation. ME‐QDs exhibited intermediate characteristics, along with an acceptable level of cytotoxicity, rapid uptake by cells, and efficient escape from lysosomes. Consequently, it is advisable to select capping agents in accordance with the specific objectives of the research.

## Graphical abstract

The effects of three different commonly employed capping agents; mercaptoethanol (ME), mercaptoacetic acid (MAA), and cysteamine (CA), on the physicochemical and optical characteristics of ZnS QDs, as well as their interactions with cells, were studied. These capping agents were found to have considerable effects on the behavior and properties of ZnS QDs such as stability, cytotoxicity and aggregation. Consequently, it is advisable to select capping agents in accordance with the specific objectives of the research.

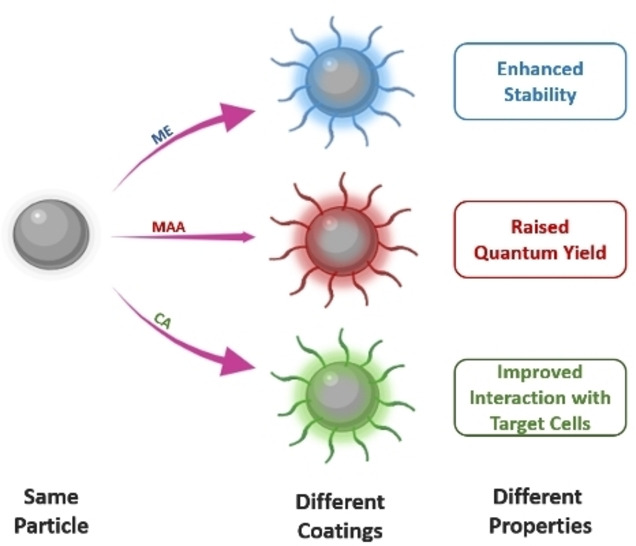

## Introduction

Quantum dots (QDs) are nearly spherical nanocrystals of semiconductor materials with sizes in the range of 2–10 nm.^[1]^ Quantum confinement effects give QDs inimitable optical, photochemical, and catalytic qualities,^[2]^ which have led to the extensive application of them especially for fluorescence sensing and bioimaging.[^3^, ^4^, ^5^] Despite the progress in generating extremely luminescent QDs, and their success in bioimaging in animals, they have yet to be successful in human body. It is recognized that QDs may interfere with various biological pathways and cause impairments in the functions of biological systems.^[6]^ Among them, zinc‐based QDs including zinc sulfide (ZnS) have shown to be eco‐friendly and biocompatible at low concentrations.^[7]^ Due to lack of toxic elements in ZnS QD, it is one of the best choices for biomedical applications.^[8]^ Nevertheless, a major obstacle in utilizing QDs for biomedical purposes is their poor colloidal stability in aqueous media.^[9]^ Surface modification of QDs with suitable capping agents can enhance their stability and keep them from aggregation.^[10]^ In addition, surface modification can raise luminescent quantum yields and increase interactions with target cells or analytes.[^11^, ^12^] Capping agents can also provide functional sites for further surface decoration with targeting moieties. So, suitable surface modification is the key point to enhance QDs colloidal and photo‐stability under biological conditions.^[8]^

Molecules having a thiol functional group may be ideal candidates for QD stabilization due to their affinity for the QD surface.^[13]^ The molecules covering the surface of a nanoparticle have an impressive effect on its fate because they may change the interface, i. e. the face of the nanoparticle that biological entities meet. They not only modify the surface chemical properties, but also can change particle size and shape, stability, optical properties, photocatalytic activities, and toxicity of nanoparticles.^[8]^ Hence, it is important to realize the differences among different capping agents and their impact on the physicochemical as well as biological properties of nanoparticles.

Numerous capping agents containing thiol groups have been employed to stabilize quantum dots (QDs) in previous studies. In this particular research, we present the synthesis and analysis of ZnS QDs stabilized using three commonly utilized capping agents: mercaptoethanol (ME), mercaptoacetic acid (MAA), and cysteamine (CA). The objective is to compare the effects of these capping agents on various aspects of the physicochemical properties and biological interactions of ZnS QDs, including their morphology, structure, particle size, colloidal stability, optical properties, cytotoxicity, and cellular uptake.

## Materials and Methods

### Chemicals and reagents

2‐mercaptoethanol (ME, 99 %) and zinc chloride (ZnCl_2_, 98 %) were purchased from Merck, Germany. Mercaptoacetic acid (MAA, 98 %) and sodium sulfide (Na_2_S, 98 %) were purchased from Acros, United States, and cysteamine (CA, 98 %) and acridine orange (AO) from Sigma‐Aldrich. MCF7 cell line was provided by Iran Pasteur Institute. Media for cell culture including DMEM medium and Fetal Bovine Serum (FBS) were purchased from Shellmax, Iran.

### Synthesis of ZnS quantum dots

ZnS QDs were synthesized via chemical precipitation process at room temperature as described by Li, et al.^[14]^ MAA, ME, and CA were utilized as capping agents in a capping agent/Zn/S molar ratio of 4 : 1 : 1. First, 10 mmol of each capping agent was individually inserted in a two‐necked flask. Then, 25 mL of 100 mM ZnCl_2_ was added to the flask dropwise while being constantly stirred and purged with nitrogen atmosphere. The pH of the mixture was adjusted to 12 for MAA and ME^[14]^ and to 7 for CA.^[15]^ Twenty five mL of 100 mM Na_2_S was then added to the mixture under vigorous stirring. The synthesis of ZnS QDs was indicated by a white precipitate that were then centrifuged, washed, and freeze‐dried before being stored at 4 °C. Energy Dispersive X‐ray (EDX) spectrum analysis (TESCAN‐Vega3) was carried out to prove the chemical composition of ZnS QDs. In addition, an inductively coupled plasma optical emission spectrometer (ICP‐OES) (Varian Vista‐PRO) was used to quantify elemental zinc in the surface modified QDs.

### Characterization of quantum dots

The hydrodynamic diameter (Z‐average) and polydispersity index (PDI) were assigned by dynamic light scattering (DLS 180°, Microtrac, Germany). Zeta‐potential was measured in water and phosphate buffer (pH 7.4) at 25 °C by Zeta‐Check (Microtrac, Germany).

Transmission electron microscopy (TEM, Philips EM 208S, Netherlands) was performed to analyze the morphology (shape) as well as size of QDs. Samples were prepared by drying droplets of the colloids on carbon copper grids.

Atomic force microscopy (AFM) (Standard, Iran) was performed in non‐tapping mode to take 3‐dimensional (3D) images and surface properties of QDs.

Fourier‐Transform Infrared (FTIR) spectrometer (Perkin‐Elmer spectrum RX1) was used in the region of 4000–400 cm^−1^ to demonstrate the synthesis of ZnS QDs and the presence of capping agents.

The crystal structure of the samples was investigated via X‐ray diffraction (XRD) using a Bruker D8Advanced Diffractometer (Germany) with a Cu Kα source (λ=1.5406 Å) in the 2Ɵ range of 20–70 degree.

Fluorescence emission spectra of ZnS QDs were recorded by a fluorescence spectrometer (Varian Cary Eclipse, Agilent). A microplate reader was used to perform ultraviolet‐visible (UV‐vis) spectroscopy analysis (Bio‐Tek Instruments Inc., USA).

### MTT assay

Mitochondrial functionality was assessed as an indicator of cell health using the 3‐(4,5‐dimethylthiazol‐2‐yl)‐2,5‐diphenyltetrazolium bromide (MTT) assay. MCF7 cells (Iran Pasteur Institute) were seeded in a 96‐well plate (1×10^4^ cells/well) overnight. Cells were then exposed to various doses (2.5–250 μg/mL) of ZnS QDs for 24 h. MCF7 cells incubated in Dulbecco's Modified Eagle Medium (DMEM) with 10 % Fetal bovine serum (FBS) were used as control. Afterwards, cells were thoroughly washed with phosphate‐buffered saline (PBS) and treated with MTT reagent (50 μg/well). After 4 hours, the media were discarded, and sodium dodecyl sulfate (DMSO) was added to each well to dissolve the formazan crystals produced by active cell mitochondria. A microplate reader was then used to measure absorbance at 570 nm. Dividing the absorbance of the samples into the control cell viability was obtained (Equation 1.^[16]^

(1) $$cell\;viability\;\%=\frac{absorbance\;of\;samples\;at\;570\;nm}{absorbance\;of\;control\;at\;570\;nm}\times 100$$

### Cellular Uptake of QDs

MCF7 cells were seeded in 24‐well plates and incubated overnight at 37 °C and 5 % CO_2_ atmosphere before exposure to 250 μg/mL of ZnS QDs. After various times of exposure (0.5, 1, 2, and 4 hours), cells were washed with PBS and stained with acridine orange (0.1 μg/mL) for 30 minutes. Cells were then fixed by 4 % paraformaldehyde. The lysosomes were tracked at 640 nm while excited at 457 nm^[17]^ using a microplate fluorimeter (Infinite 200, Tecan, Austria).

## Results and Discussion

The effects of various capping agents on the physicochemical and biological characteristics of ZnS QDs were compared. The ZnS QDs were extensively characterized using spectroscopic techniques (FTIR, UV‐vis, and PL), and their size and shape were investigated using DLS and TEM, as well as their structural features using XRD. The biological interaction of differently coated QDs was also studied with in vitro cytotoxicity tests.

### pH Effect on synthesis process of quantum dots

Studies have shown that the quantum yield of QDs depends on the pH at which they are synthesized.[^14^, ^18^] Li et al. showed that more alkaline conditions (12 vs. 8) were more favorable for synthesis of ZnS QDs. They concluded that lower solubility of ZnS leads to better crystallization at higher pH.^[14]^ However, our experience showed that the pH effect also depends on the pKa of capping agent, as it dictates the ionic state of each capping agent at any given pH. The high pH was not favorable for the CA‐stabilized QDs, as the materials precipitated quickly before they could form nanocrystals. It shows that the ionic state of capping agent is as important as the solubility of ZnS crystals. The thiol group of the capping agent has affinity for the surface of nanocrystals. The lower thiol p*K*_a_ of CA compared to the other two capping agents (Table 1) caused more proportion of the thiol groups of CA to convert to negatively charged thiolates at pH 12 of the reaction mixture, which could result in formation of uncoated particles that precipitated as aggregates.^[15]^ Yaraki et al. have also shown that the optimum pH for synthesis of CA stabilized QDs is 7.^[15]^

**Table 1:** Attributes of the used capping agents.

Name	Chemical Structure	Molecular formula	p*K*_a_ (at 25 °C)
Mercaptoethanol	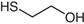	HOCH_2_CH_2_SH	9.72
Mercaptoacetic acid	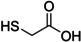	HSCH_2_COOH	p*K*_a1_=3.6 (carboxyl) p*K*_a2_=10.5 (thiol)
Cysteamine	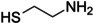	[HSCH_2_CH_2_NH_3_]^+^cl	p*K*_a1_=8.19 (thiol) p*K*_a2_=10.75 (amine)

The EDX spectra of QDs confirmed the presence of the elements Zn, S, C, O, and N (in the case of CA QDs) by the appearance of corresponding peaks in the sample (Figure S1).

**Figure 1:**
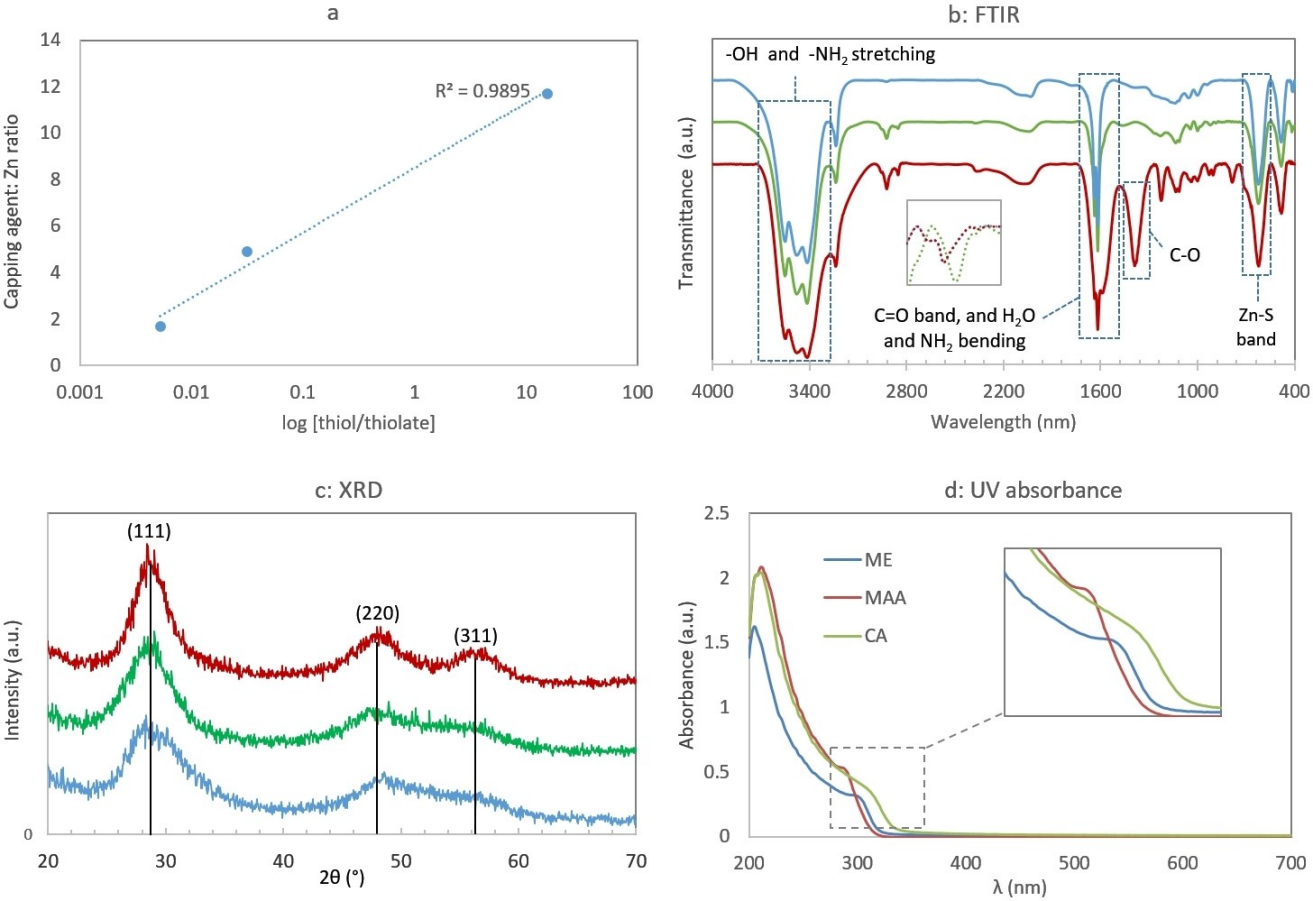
Characteristics of ZnS QDs; a: correlation between the concentration ratio of non‐ionized to ionized SH group and the molar ratio of capping agent to Zn atoms, b to d: FTIR, X‐ray diffraction, and UV‐visible absorbance spectra of differently coated QDs; the inset in b shows the bands related to stretching vibration of S−H bond in thiol groups of free capping agents. ME: mercaptoethanol‐, MAA: mercaptoacetic acid‐, CA: cysteamine‐coated ZnS QDs

Inductively coupled plasma (ICP) spectroscopy was carried out to determine the amount of zinc element in the prepared coated QDs. Since the ratio of Zn to S in the ZnS nanocrystals is 1 : 1 (look at crystal structure), measuring the amount of zinc also determines the amount of sulfur. Similarly, the amount of capping agent could be estimated. The measured content of Zn based on ICP spectroscopy and calculated contents of S and different capping agents for 10 mg/L of stabilized QDs is shown in Table S1. As it is indicated in the table the final molar ratio of capping agent to Zn (and S) varies greatly from 1.7 for ME to 4.9 for MAA and 11.7 for CA. According to their different p*K*_a_, the different behavior of capping agents could be attributed to the degree of their thiol ionization and the ratio of thiol to thiolate at different pHs (Table S1).^[15]^ As shown in Figure 1a, the coverage of QD surface (indicated by capping agent to Zn molar ratio) has direct correlation with logarithm of thiol to thiolate concentration ratio (R^2^=0.989). The results show that the higher the non‐ionized thiol proportion (the ratio of thiol to thiolate) the more efficient the QD surface coverage. In addition, the solubility of ZnS increases as the pH decreases. More solubility hinders the elements from crystal formation and results in lower ZnS content. So, it seems that pH is very critical to make a balance between thiol ionization and ZnS solubility.

Regarding the effects of coating thickness on the stability as well as optical properties of QDs it would be beneficial to separately optimize the initial molar ratio of any capping agent for QD synthesis.

### Characterization of Quantum Dots

#### Morphology and Particles Size

As expected, capping agents affected particle size and zeta potential of synthesized QDs (Table 2). The histograms of size distribution of ZnS QDs determined by DLS showed monodisperse distributions with an average hydrodynamic diameter of 4.93, 10.51, and 53.5 nm for ME‐, MAA‐, and CA‐QDs respectively (Figure S2). The surface charge of ZnS QDs in water was negative for ME‐ and MAA‐QDs and positive for CA‐QDs as expected, which clearly shows the interactions of capping agent with QDs (Table 2).

**Table 2:** Characteristics of differently coated ZnS QDs and comparison of their size determined by different methods (nm).^[a]^

ZnS QD Characteristics		Capping agents		
		MAA	ME	CA
Zeta potential (mV)	In deionized water	−51.25±0.64	−40.95±1.20	−33±0.99
	In phosphate buffer (pH 7.4)	−35.65 ±0.92	−28.95±1.63	−24.25±1.06
Capping agent : Zn	Molar ratio	4.9	1.7	11.7
UV absorbance (nm)		285	295	297
Bang gap energy (eV)		4.356	4.209	4.180
Relative quantum yield		1	0.89	0.28
β (radian)	Full width at half‐maximum	0.064	0.129	0.090
Strain	β/4tanθ	0.062	0.125	0.088
Dislocation density	15 ϵ/aD	0.071	0.352	0.148
Size (nm)	DLS (PDI)	10.51 (0.16)	4.93 (2.3)	53.5 (0.29)
	TEM	6.8	6	ND
	UV spectroscopy	2.78	3.02	3.07
	XRD: Scherrer Equation	2.32	1.16	1.65
	XRD: Modified Scherrer eq.	2.57	1.04	1.74
	XRD: Williamson‐Hall method	4.02	0.89	2.32

[a]MAA: Mercaptoacetic acid; ME: mercaptoethanol; CA: cysteamine; ND: not determined.

**Figure 2:**
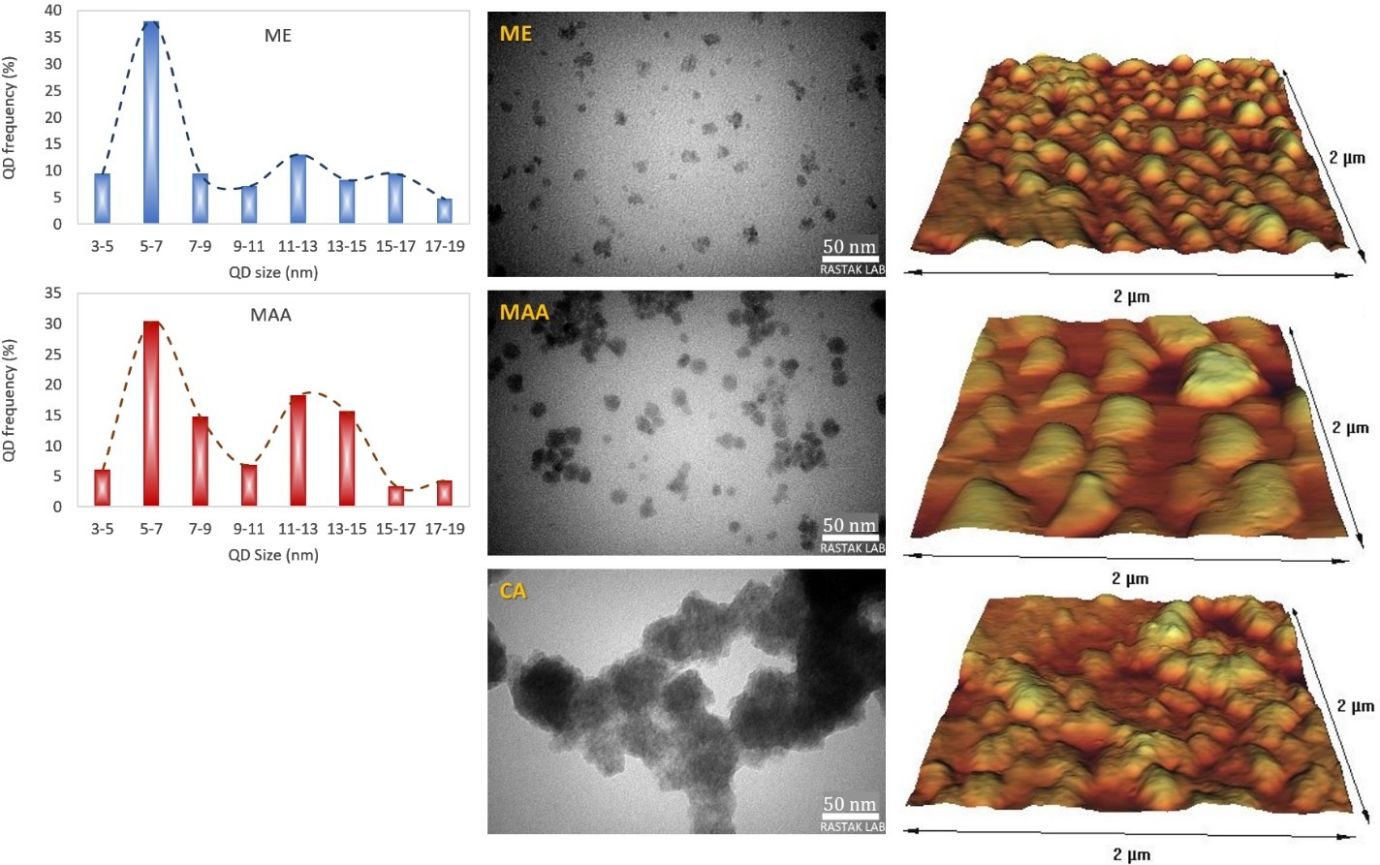
Histogram of QD size distribution (left) obtained from TEM images (middle), in comparison with their 3D AFM images (right). ME: mercaptoethanol‐, MAA: mercaptoacetic acid‐, CA: cysteamine‐coated ZnS QDs

TEM images showed spherical nanoparticles with a considerable degree of agglomeration (Figure 2). It seems that these stabilized QDs tend to aggregate during drying process for microscopy. ME‐QDs had the lowest degree of aggregation. CA‐QDs were completely aggregated so that it was not easy to distinguish particle boundaries. Histogram of size distribution showed particle sizes about 6 and 6.8 nm for ME‐and MAA‐QDs, respectively. The lower particle size of ME‐QDs acquired by DLS indicates that the majority of them exist as discrete QDs in colloidal state, whereas drying causes their aggregation, as mentioned earlier.

The AFM analysis showed that agglomeration caused a decrease in the roughness of the samples due to the increase in grain size (Figure 2), so that the highest roughness was recorded for ME‐QDs, which were shown to have the lowest agglomeration (Table S2). Gupta et al. have also reported the inverse relationship between nanoparticle size and the roughness of dried samples obtained by AFM.^[19]^

#### Fourier‐Transform Infrared Spectroscopy (FTIR)

FTIR spectroscopy was carried out to assess the chemical groups existing on the surface of ZnS QDs and the interactions between the functional groups of the capping agents and the ZnS QDs (Figure 1b). The FTIR spectrum of free CA displays the stretching vibration band of S−H group around 2500 cm^−1^, which was almost disappear in the FTIR spectrum of CA‐QDs, confirming the covalent binding of thiol group to the surface of QDs and no free CA in the colloid.^[15]^ The absorption bands at 3200–3500 cm^−1^ are related to stretching vibration of O−H (H_2_O) and N−H (−NH_2_). The sharp band at 1651 cm^−1^ can be due to the bending of the NH_2_ group on the surface of QDs. A strong band at 623 cm^−1^ is remarkable for Zn−S (metal sulfide) bond.

The FTIR spectrum of ME‐QDs demonstrate absorption bands at 3480 cm^−1^ related to stretching vibration of OH. Absorption band of the −SH group at 2569 cm^−1^ disappeared again in ME‐QDs, which is related to the cleavage of S−H bond due to the covalent interaction with QDs. The band at 1138 cm^−1^ is related to the C−O stretching, and a remarkable band demonstrated at 623 cm^−1^ is related to ZnS QDs.

Similarly, the FTIR spectrum of MAA‐ ZnS QDs showed a strong and broad band in the region 3482 cm^−1^, which is related to O−H. The observed bands around 1587 cm^−1^ (C=O band) and 1225 cm^−1^ (C−O band) are attributed to the MAA carboxylic acid group. The strong band at 622 cm^−1^ shows the Zn−S bond in QDs. However, there was no obvious band at 2568 cm^−1^, related to S−H bond, confirming the attachment of MAA to the surface of QDs via the thiol group, protruding the carboxylic acid groups to the medium. Overall, the FTIR results demonstrated that the capping agents successfully modified the surface of ZnS QDs.

### X‐Ray Diffraction Analysis

#### Crystal structure

XRD analysis shows 3 specific peaks at 2θ almost equal to 29, 48, and 56° which are related to the reflection from (111), (220), and (311) sheets respectively, the typical pattern for ZnS QDs according to the literature^[20]^ (Figure 1c). Having the Miller indices (h k l), lattice constants (i. e., the unit cell edges a, b, c) can be extracted from Equation 2:

(2) $$\;\frac{1}{d^{2}}=\frac{h^{2}}{a^{2}}+\frac{k^{2}}{b^{2}}+\frac{l^{2}}{c^{2}}$$

where d is the space between the crystal layers (or path difference) obtained from Bragg's law: d=λ/2sinθ, which is shown in Table S3 for each sheet. The calculation of lattice constant for the MAA showed that all lattice constants are almost equal (a=b=c≈5.3 Å) which means that the structure of crystals is cubic which is in accordance with literature.^[21]^ ZnS atoms may arrange in cubic or hexagonal crystalline structures. The cubic phase also known as zinc blend is more stable at room temperature and consists of 1 : 1 Zn to S ratio.^[22]^

#### Crystallite Size Determination

The Scherrer equation is utilized to calculate the crystallite size D from XRD data, which is equal to Kλ/βCosθ (Equation 3). Where K is a shape constant that can be 0.62–2.08 and is usually taken as about 0.9, λ is the wavelength of the X‐ray in nanometer (0.154056 nm for Cu K_ɑ_ radiation), β is the “full width at half‐maximum intensity” of the peak (FWHM), and θ is the peak position.

(3) $$D=\frac{K\lambda }{\beta \cdot cos\theta }$$

To exclude the systematic error in the Scherrer equation that results in different size estimates based on different peaks, Monshi et al. introduced a modified version of the Scherrer equation to calculate the nanocrystallite size through an average value of any number of peaks (Equation 4). 4

(4) $$ln\;\beta =ln\;\frac{K\lambda \;}{D}+ln\;\frac{1}{cos\theta }$$

**Figure 3:**
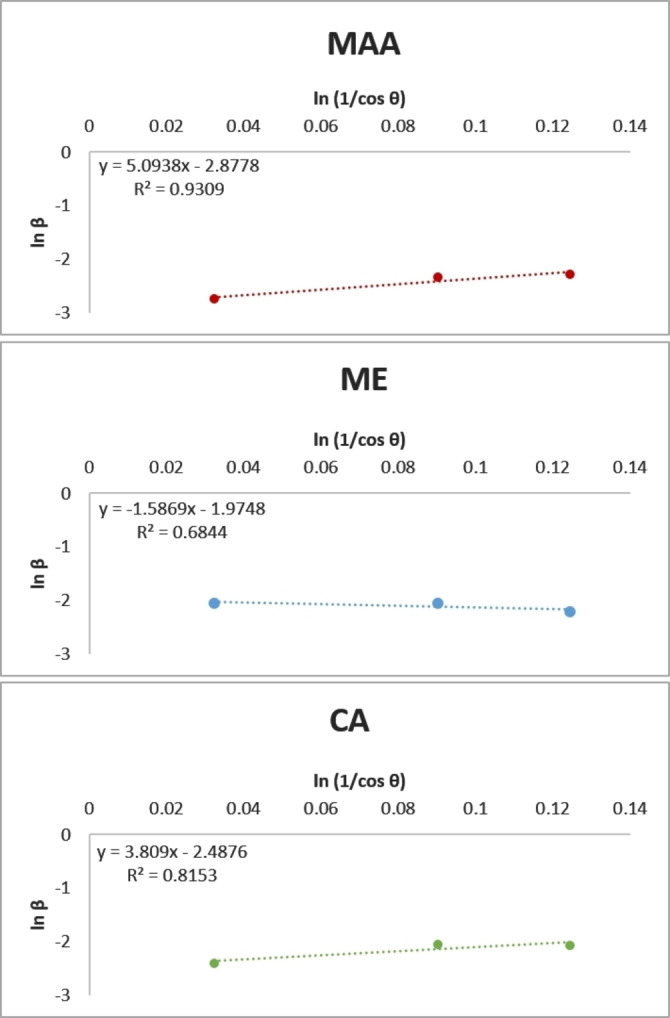
Modified Scherrer equation plots, the intercept of the lines equals lnKλ/D . ME: mercaptoethanol‐, MAA: mercaptoacetic acid‐, CA: cysteamine‐coated ZnS QDs

Based on the modified Scherrer equation, plots were drawn with ln (1/cosθ) on the x‐axis and ln β along the y‐axis (Figure 3). The intercept of the straight line is lnKλ/D from which D was calculated (Table 2).

In addition to crystallite size, the XRD peak width can also indicate the lattice microstrain, hence the peak broadening may show more strain in the crystal lattice, which may be resulted from dislocations or crystal imperfections.^[23]^ Therefore, the broadening of peaks in (220) and (311) sheets may indicate crystal lattice imperfection in ME and CA QDs. This imperfection affects the peak width at half maximum intensity (FWHM) which is used to calculate crystallite size. For this reason, using XRD data may cause underestimation of particle size. As shown in Table 2, the calculated size for MAA was closer to the size estimated by the UV spectroscopy method, while for the other two QDs, the XRD calculated sizes were much smaller. The lattice strain can be obtained from the Equation 5:

(5) $$Strain\;\left(\epsilon \right)=\frac{\beta }{4\tan\theta }$$

According to the Williamson‐Hall method, β of a specimen is the result of both size and microstrain, so the equation changes to Equation 6:

(6) $$\beta \cdot cos\theta =\;\frac{K\lambda }{D}+4\epsilon \cdot sin\theta$$

When β ⋅ cosθ was plotted vs. sinθ, size and strain could be calculated from intercept and slope of the linear regression respectively (Figure S3). Nevertheless, as indicated in Table 2, the sizes determined by the Williamson‐Hall method were inconsistent with previous results and do not appear to be acceptable for estimating the size of these QDs.

### Optical Characterization

#### Fluorimetry and Quantum Yield Measurement

**Figure 4:**
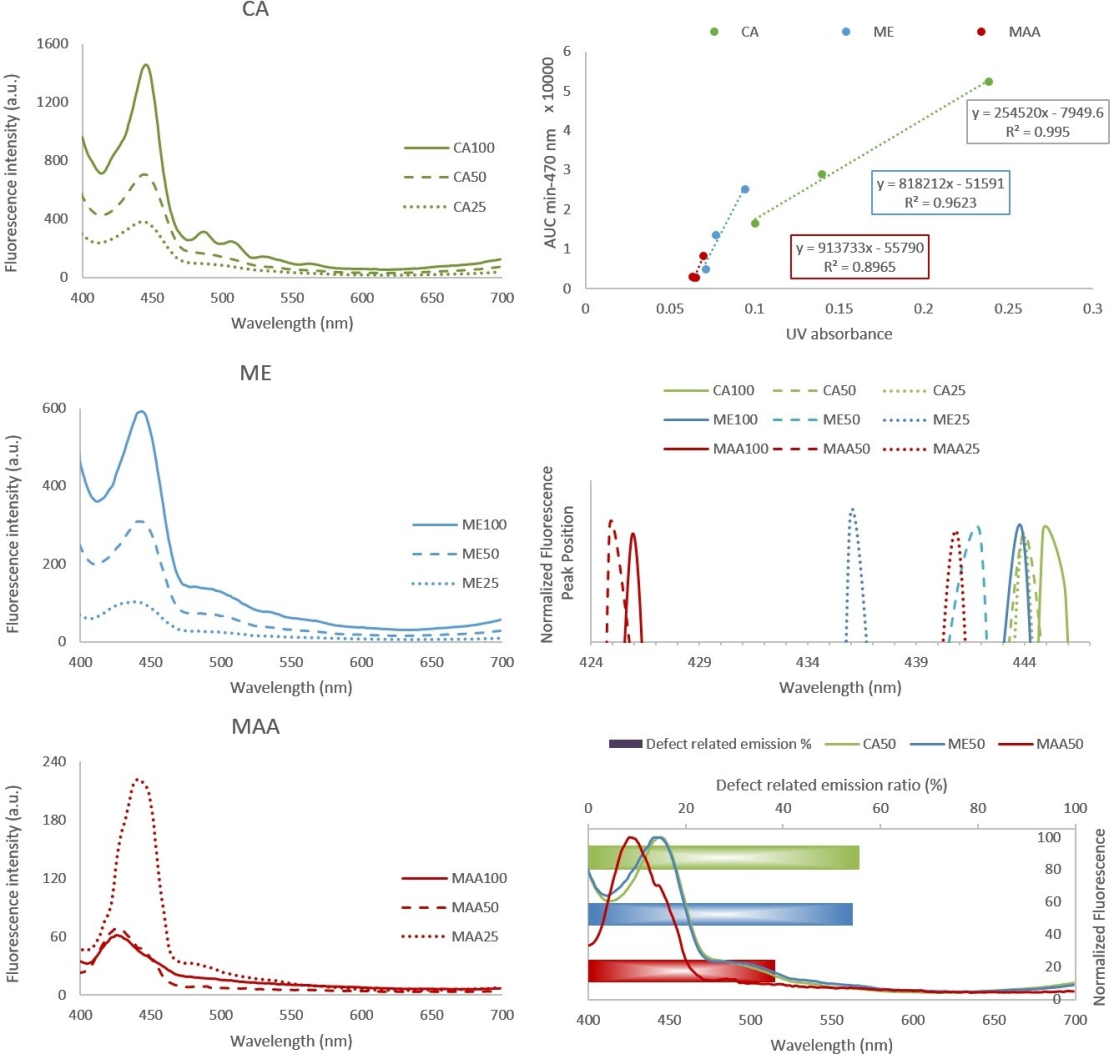
Left: Fluorescence emission spectra of differently coated ZnS QDs at different concentrations. Upper right: Integrated fluorescence intensity vs. UV absorbance, the slope of each line shows the relative quantum yield. Middle right: Peak position of normalized fluorescence spectra. Lower right: defect‐related emission in normalized spectra, bars show the ratio of defect ‐related AUC to the whole emission AUC. ME: mercaptoethanol‐, MAA: mercaptoacetic acid‐, CA: cysteamine‐coated ZnS QDs. The numbers in sample names show the QD concentration in μg/mL.

The fluorescent properties of QDs including emission wavelength and quantum yield depends on their size and crystal structure which in turn could be affected by capping agent used during synthesis. The fluorescence emission of all QDs was mostly excited at 370 nm with maximum emission around 444 nm. The fluorescence spectrum of QDs at lower concentration (25 μg/mL) showed a red shift in order of ME < MAA < CA which is in accordance with DLS and TEM results. However, MAA‐QDs showed a considerable blue shift at higher concentrations (≥50 μg/mL) (Figure 4)

The fluorescence intensities of CA and ME QDs were concentration‐dependent, and they showed a red shift with increasing concentration, which was more noticeable for ME QDs. Regarding the zeta potential of the stabilized QDs, they are assumed more discrete at lower concentrations, while as the concentration increases particles tend to assemble. The optoelectronic properties of QDs are affected by that; wave function overlap, electronic coupling, and charge transfer have been reported for QD oligomers.^[24]^ Excitation energy transfer can take place within QD oligomers, where the electronic energy is transferred from a smaller particle with larger bandgap to a larger particle with a smaller bandgap, resulting in a red shift of emission spectra.[^24^, ^25^]

However, the fluorescence intensity of MAA QDs did not follow the pattern of the other two QDs. While the intensity remained unchanged as the concentration decreased from 100 to 50 μg/mL, it steeply raised more than 3‐fold with a red shift of about 16 nm at 25 μg/mL. This may be due to self‐quenching at higher concentrations as similarly reported for other QDs,[^26^, ^27^] but the threshold concentration may vary depending on the capping agent used. Regarding the blue shift of MAA‐QDs, it is plausible that agglomeration of these QDs at higher concentrations primarily involves larger particles, resulting in a shift of the fluorescence emission spectrum to the blue wavelength region.

The emissions above 470 nm are related to defects in crystal lattice where the excitation energy transfers from donors to defect states resulting in a tail in emission spectrum.^[25]^ Higher defect‐related emission in ME‐ and CA‐QDs shows more imperfection compared to MAA‐QDs (Figure 4) as it was also shown in XRD.

Quantum yield (QY) shows the efficiency of a fluorophore in transforming the exciting photons into the emitting photons. Conventionally, QY of a fluorescent material can be obtained using the Equation 7.

(7) $$Q=Q_{r\;}\times \frac{I}{I_{r}}\times \frac{A_{r}}{A}\times \frac{n^{2}}{n_{r}^{2}}$$

Where *Q* is the QY, *I* is the integrated fluorescence intensity, *A* is the absorbance at the excitation wavelength, *n* is the refractive index of the medium, and the subscript *r* refers to the reference fluorophore of known quantum yield.^[28]^ According to Williams’ and Winfield method, plotting the integrated intensity of the fluorescence spectra at different concentrations vs. absorbance of the sample at the same concentration yields a line whose slope shows the relative quantum yield (rQY),^[29]^ which is very useful for comparison of QYs of different samples (Figure 4). Since UV absorbance is a function of QD concentration, dividing the fluorescence by the UV absorbance also corrects for the effect of the difference in concentration of ZnS in stabilized QDs. Although CA‐QDs showed the strongest fluorescence intensity, followed by ME and MAA, their rQY were in reverse order, with MAA QDs having the highest rQY (Table2).

#### Band Gap Energy Analysis

UV‐visible spectrometry can be applied to assess the band gap energy of QDs; the minimum energy that the valence band electrons require exciting up to the conduction band. The band gap energy can be determined based on the famous equation E=hν, where ν=C/λ (Equation 8) 8

(8) $$Energy\;\left(E\right)=Planks\;Constant\;\left(h\right)\times \frac{Speed\;of\;Light\;\left(C\right)}{Wavelength\;\left(\lambda \right)}$$

The UV‐visible spectra of ZnS QDs showed excitonic transitions (λ_exc_) around 300 nm (Figure 1d). The blue shift from bulk values (388 nm for bulk ZnS)^[30]^ shows the construction of QDs. To accurately determine the characteristic absorbance wavelength (λ_exc_), the derivative of absorbance was calculated as the central finite difference at each point. As expected, the band gap values were greater than value of bulk ZnS (3.68 eV)^[31]^ (Table 2).

Band gap energy was then employed to calculate the QD size according to the Equation 9:

(9) $$D=\frac{0.32-\left(2.9\;\sqrt{E_{g\;}-3.49}\;\right)}{3.50-E_{g\;}}$$

where, E_g_ is the band gap energy in eV and D is the size of the nanoparticle in nm.^[32]^ The calculated sizes for all three differently stabilized QDs was almost the same, around 3 nm with an order of MAA<ME<CA that was in accordance with fluorimetry results. However, it is worth noting that the size calculated here is related to nanocrystallites and may not necessarily correspond to the size of nanoparticles present in the colloid, as the nanoparticles may be found in the form of dimers or oligomers due to self‐assembly.

### Biological Interactions

The surface coating and functionalization can significantly affect the interaction of nanoparticles with their surroundings, including biological entities; because what the cells are facing is not the bare surface of nanoparticles itself, but the molecules that enfold it. Hence, when assessing a nanoparticle for biomedical purposes, it is very essential to study the impact of coating material on its biological interactions. Cytotoxicity of nanoparticles as well as the way and the extent of their internalization into cells are among the most important biological interactions which should be assessed before any biomedical application.

### MTT Assay

**Figure 5:**
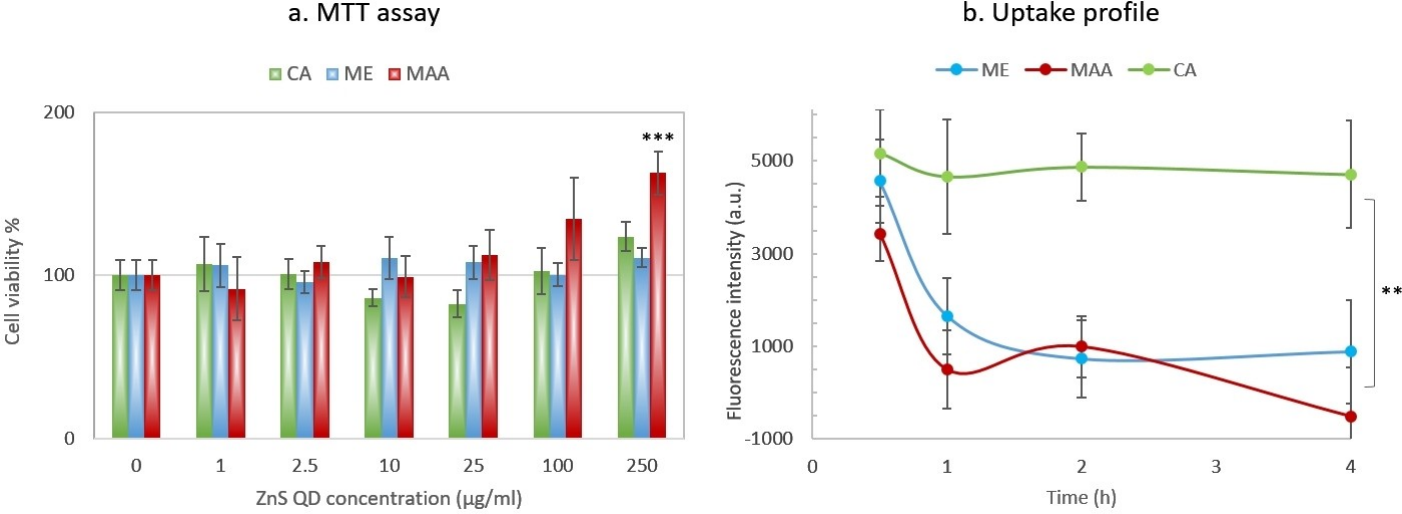
Biological interactions of differently (mercaptoethanol (ME), mercaptoacetic acid (MAA), cysteamin (CA)) coated ZnS QDs

To evaluate the effect of different capping agents on the biological interactions of QDs, their cytotoxicity was studied by MTT assay. None of the QDs showed significant cytotoxicity in concentrations up to 250 μg/mL (Figure 5a), which is in accordance with another study on L929 mouse fibroblast cells with D‐glucose stabilized ZnS QDs.^[33]^ Nevertheless, higher concentrations of MAA QDs showed a trend of proliferation induction, so that there was a significant difference with the untreated control at the 250 μg/mL concentration. This difference could originate from the difference in the amount of internalized QDs or to the toxicity of the capping molecules. An in vivo study carried out by National Toxicology Program (NTP) showed that a high dose of MAA (360 mg/kg) can cause “a significant increase in the number” of peripheral blood cells, but only in female mice.^[34]^ Other studies have also shown the role of cell sex in cytotoxicological assays.^[35]^ However, the studies are contentious, and the cellular uptake rate and extent should be assessed that will be explained in the next part.

### Cellular Uptake

The interaction of nanoparticles with cells has a direct effect on their uptake, which in turn influences the subsequent biological impacts of nanoparticles, including cytotoxicity. Direct analysis of QD uptake by measuring their own fluorescence may be misleading since a significant fraction of nanoparticles adhere to the surface of the cells and are not simply washed away.^[36]^ In this case, the interpretation of results based on nanoparticle emission would result in overestimation of nanoparticle uptake. In addition, it cannot imply the uptake mechanism. On the other hand, since the main route of cell entry for nanoparticles is endocytosis,^[37]^ indirect assessment of uptake by tracking cellular lysosomes tells us not only about the extent and rate of uptake, but also about what happens after the cell membrane is crossed.^[38]^ QDs have been shown to enter cells by endocytosis as well, leading to particle transport to late endosomes or lysosomes.^[39]^ Due to its weak basic property, acridine orange is accumulated in acidic environment of lysosomes and its fluorescence emission shifts from green to red wavelength region (640 nm).

It seems that the QDs are taken up rapidly at the first 30 minutes of exposure to the cells (Figure 5b). There was no significant difference between the extents of cellular uptake of different QDs. However, while the number of lysosomes remained almost constant over the next 3.5 h in CA‐QDs, it rapidly decreased in the other two groups (MAA‐ and ME‐QDs). The rapid decline in cell lysosome content may indicate a destabilization of lysosomal membrane due to interaction with nanoparticles, which probably resulted in lysosome degradation and nanoparticle escape. The values of capping agents (ME, MAA, and CA) indicate that they will remain neutral, anionic, and cationic respectively in the pH of lysosomes. So, the membrane destabilization could not be simply attributed to the charge. Some studies have shown the effect of CA on the inhibition of lysosomal enzyme activity.[^40^, ^41^] Enzyme inhibition may contribute to increase in the retention time of nanoparticles inside the lysosomes. However, understanding the exact underlying mechanism needs more investigations.

## Conclusions

Biomedical application of inherently hydrophobic QDs (1) requires capping agents that stabilize their aqueous dispersions, also called stabilizing agents. The stabilizing agent used during synthesis process of QDs has a crucial effect on the physicochemical, optoelectrical, and biological properties of QDs. Hence, choosing an appropriate capping agent is an essential step in biomedical studies. It seems that there is no versatile, one‐size‐fits‐all capping agent; each has its own pros and cons and should be picked with respect to the purpose.

Regarding the findings obtained by different characterization methods, it may be concluded that unlike ME‐QDs, MAA and CA‐QDs are rarely found as single nanocrystals but mainly as dimers or oligomers, especially at higher concentrations. Although MAA‐QD showed a more perfect crystal lattice with smaller size and considerable QY, its biological effect was not so favorable. In addition, its overall poor absorption at 370 nm makes it less luminescent compared to the two other QDs, which along with its lower biocompatibility makes it less desirable for bioimaging purposes. CA‐QDs, on the other hand, showed the lowest QY and probably lysosomal enzyme inhibition, which hinders nanoparticles from escaping from lysosomes. Moreover, transmission electron microscopy showed that they also tend to agglomerate more than the other two QDs, which is associated with their lower colloidal stability and shorter shelf life. While CA‐QDs exhibit favorable interaction with cell membranes due to their positive charge at physiological pH, this characteristic often results in their cell attachment and consequently can interfere with subsequent analyses, including intracellular assays. Nonetheless, this feature can prove beneficial for imaging the cell surface. On the other hand, ME‐QDs displayed intermediate properties and cell behavior. Although their crystal lattice was not as flawless as that of MAA‐QDs, this imperfection does not appear to pose a significant issue, as ME‐QDs efficiently emit fluorescent light. Furthermore, they showed an acceptable cytotoxicity profile, rapid cellular uptake, and effective lysosomal escape, which besides appropriate colloidal stability make them good candidates for bioimaging applications.

## Supporting Information Summary

The Supporting Information includes further analysis results including EDX and size distribution graphs as well as some calculations including the capping agent to ZnS ratio, surface roughness measured by AFM, and d spacing estimated by Brag's law.

## Conflict of interest

There is no conflict of interest to disclose.

1

## Supporting information

As a service to our authors and readers, this journal provides supporting information supplied by the authors. Such materials are peer reviewed and may be re‐organized for online delivery, but are not copy‐edited or typeset. Technical support issues arising from supporting information (other than missing files) should be addressed to the authors.

Supporting Information

## Data Availability

The data that support the findings of this study are available from the corresponding author upon reasonable request.

## References

[1] B. Gidwani , V. Sahu , S. S. Shukla , R. Pandey , V. Joshi , V. K. Jain , A. Vyas , J. Drug Delivery Sci. Technol. 2021, 61, 102308.

[2] C. Zhu , Z. Chen , S. Gao , B. L. Goh , I. B. Samsudin , K. W. Lwe , Y. Wu , C. Wu , X. Su , Prog. Nat. Sci. 2019, 29, 628–640.

[3] A. A. Mansur , F. G. de Carvalho , R. L. Mansur , S. M. Carvalho , L. C. de Oliveira , H. S. Mansur , Int. J. Biol. Macromol. 2017, 96, 675–686. 28049016 10.1016/j.ijbiomac.2016.12.078

[4] D. Geißler , L. J. Charbonnière , R. F. Ziessel , N. G. Butlin , H. G. Löhmannsröben , N. Hildebrandt , Angew. Chem. Int. Ed. 2010, 49, 1396–1401. 10.1002/anie.200906399 20108296

[5] P. Zrazhevskiy , M. Sena , X. Gao , Chem. Soc. Rev. 2010, 39, 4326–4354. 20697629 10.1039/b915139g PMC3212036

[6] O. A. Aladesuyi , T. C. Lebepe , R. Maluleke , O. S. Oluwafemi , Nanotechnol. Rev. 2022, 11, 2304–2319.

[7] H. R. Rajabi , M. Farsi , J. Mol. Catal. A 2015, 399, 53–61.

[8] H. R. Rajabi , M. Farsi , Mater. Sci. Semicond. Process. 2016, 48, 14–22.

[9] A. M. Wagner , J. M. Knipe , G. Orive , N. A. Peppas , Acta Biomater. 2019, 94, 44–63. 31082570 10.1016/j.actbio.2019.05.022 PMC6642839

[10] A. Kortan , R. Hull , R. L. Opila , M. G. Bawendi , M. L. Steigerwald , P. Carroll , L. E. Brus , J. Am. Chem. Soc. 1990, 112, 1327–1332.

[11] C. J. Murphy , J. Anal. Chem. 2002, 74, 520 A-526 A.

[12] N. P. Gandhi , J. V. Rohit , M. A. Kumar , S. K. Kailasa , Res. Chem. Intermed. 2013, 39, 3631–3639.

[13] M. Green , J. Mater. Chem. 2010, 20, 5797–5809.

[14] H. Li , W. Y. Shih , W.-H. Shih , Nanotechnology. 2007, 18, 205604.

[15] M. T. Yaraki , M. Tayebi , M. Ahmadieh , M. Tahriri , D. Vashaee , L. Tayebi , J. Alloys Compd. 2017, 690, 749–758.

[16] S. Kamiloglu , G. Sari , T. Ozdal , E. Capanoglu , Food Front. 2020, 1, 332–349.

[17] G. Di Pompo , K. Kusuzaki , M. Ponzetti , V. F. Leone , N. Baldini , S. Avnet , Biomedicine 2022, 10, 1904. 10.3390/biomedicines10081904 PMC9405350 36009451

[18] J. Kim , B. T. Huy , K. Sakthivel , H. J. Choi , W. H. Joo , S. K. Shin , M. J. Lee , Y.-I. Lee , Sens. Bio-Sens. Res. 2015, 3, 46–52.

[19] S. Gupta , P. Brouwer , S. Bandyopadhyay , S. Patil , R. Briggs , J. Jain , S. Seal , J. Nanosci. Nanotechnol. 2005, 5, 1101–1107. 16108434 10.1166/jnn.2005.151

[20] A. F. Mohammed , W. R. Salah , J. Phys.: Conf. Ser, IOP Publishing 2018, pp. 012010.

[21] C. Liu , Y. Ji , T. Tan , J. Alloys Compd. 2013, 570, 23–27.

[22] H. Labiadh , S. Hidouri , J. King Saud Univ. Sci. 2017, 29, 444–450.

[23] T. Ungár , Scr. Mater. 2004, 51, 777–781.

[24] X. Xu , S. Stöttinger , G. Battagliarin , G. Hinze , E. Mugnaioli , C. Li , K. Müllen , T. Basché , J. Am. Chem. Soc. 2011, 133, 18062–18065. 22003956 10.1021/ja2077284

[25] R. Koole , P. Liljeroth , C. de Mello Donegá , D. Vanmaekelbergh , A. Meijerink , J. Am. Chem. Soc. 2006, 128, 10436–10441. 16895408 10.1021/ja061608w

[26] P. Liao , Z.-Y. Yan , Z.-J. Xu , X. Sun , Spectrochim. Acta Part A 2009, 72, 1066–1070. 10.1016/j.saa.2008.12.039 19201257

[27] M. Shamsipur , H. R. Rajabi , Mater. Sci. Eng. C. 2014, 36, 139–145. 10.1016/j.msec.2013.12.001 24433896

[28] J. Hu , C.-y. Zhang , Anal. Chem. 2013, 85, 2000–2004. 23379689 10.1021/ac3036487

[29] A. T. R. Williams , S. A. Winfield , J. N. Miller , Analyst. 1983, 108, 1067–1071.

[30] K. Rajeshwar , N. R. de Tacconi , C. Chenthamarakshan , Chem. Mater. 2001, 13, 2765–2782.

[31] J. Borah , K. Sarma , Acta Phys. Pol. 2008, 114, 713–719.

[32] D. Ayodhya , M. Venkatesham , A. S. Kumari , K. G. Mangatayaru , G. Veerabhadram , J. Appl. Chem. 2013, 6, 101–109.

[33] J. M. Baruah , S. Kalita , J. Narayan , Int. Nano Lett. 2019, 9, 149–159.

[34] M. Mercado-Feliciano , M. J. Hooth , A. Nyska , J. B. Bishop , C. R. Blystone , R. S. Chhabra , K. J. Cimon , P. M. Foster , A. P. King-Herbert , G. E. Kissling , L. L. Lanning , D. E. Malarkey , B. S. McIntyre , D. R. Ragland , H. Seung , C. S. Smith , G. S. Travlos , J. C. Turnier , M. K. Vallant , S. Waidyantha , N. J. Walker , M. L. Wenk , K. L. Witt , G. W. Wolfe , Toxic. Rep. Ser. 2016, 80, DOI: 10.22427/NTP-TOX-80. PMC8040344 33530656

[35] V. Serpooshan , S. Sheibani , P. Pushparaj , M. Wojcik , A. Y. Jang , M. R. Santoso , J. H. Jang , H. Huang , R. Safavi-Sohi , N. Haghjoo , ACS Nano 2018, 12, 2253–2266. 29536733 10.1021/acsnano.7b06212

[36] A. M. Alkilany , N. N. Mahmoud , F. Hashemi , M. J. Hajipour , F. Farvadi , M. Mahmoudi , Chem. Res. Toxicol. 2016, 29, 943–948. 27249426 10.1021/acs.chemrestox.6b00108

[37] B. Yameen , W. I. Choi , C. Vilos , A. Swami , J. Shi , O. C. Farokhzad , J. Controlled Release. 2014, 190, 485–499. 10.1016/j.jconrel.2014.06.038 PMC4153400 24984011

[38] F. Farvadi , M. H. Ghahremani , F. Hashemi , M. R. Hormozi-Nezhad , M. Raoufi , S. Zanganeh , F. Atyabi , R. Dinarvand , M. Mahmoudi , J. Colloid Interface Sci. 2018, 531, 245–252. 30032011 10.1016/j.jcis.2018.07.013

[39] L. W. Zhang , N. A. Monteiro-Riviere , Toxicol. Sci. 2009, 110, 138–155. 19414515 10.1093/toxsci/kfp087

[40] T. Jeitner , J. Oliver , J. Endocrinol. 1990, 125, 75–80. 2110966 10.1677/joe.0.1250075

[41] Y. Wen , F. Ahmad , Z. Mohri , P. D. Weinberg , D. S. Leake , Atherosclerosis. 2019, 291, 9–18. 31629988 10.1016/j.atherosclerosis.2019.09.019 PMC6912160

