# Supplementary material for: Exploring the Effects of Various Capping Agents on Zinc Sulfide Quantum Dot Characteristics and In‐vitro Fate
Source: ChemistryOpen. 2023 Oct 6;12(10):e202300094. doi: 10.1002/open.202300094 (PMC10558426; doi:10.1002/open.202300094)
Supplement: Supplementary file 1 — Supporting Information [file OPEN-12-e202300094-s001.pdf]

ChemistryOpen

Supporting Information

Exploring the Effects of Various Capping Agents on Zinc Sulfide Quantum Dot Characteristics and In-vitro Fate

Zohre Montaseri, Ali Mohammad Tamaddon,* Mohammad Javad Raee, and Fakhrossadat Farvadi*

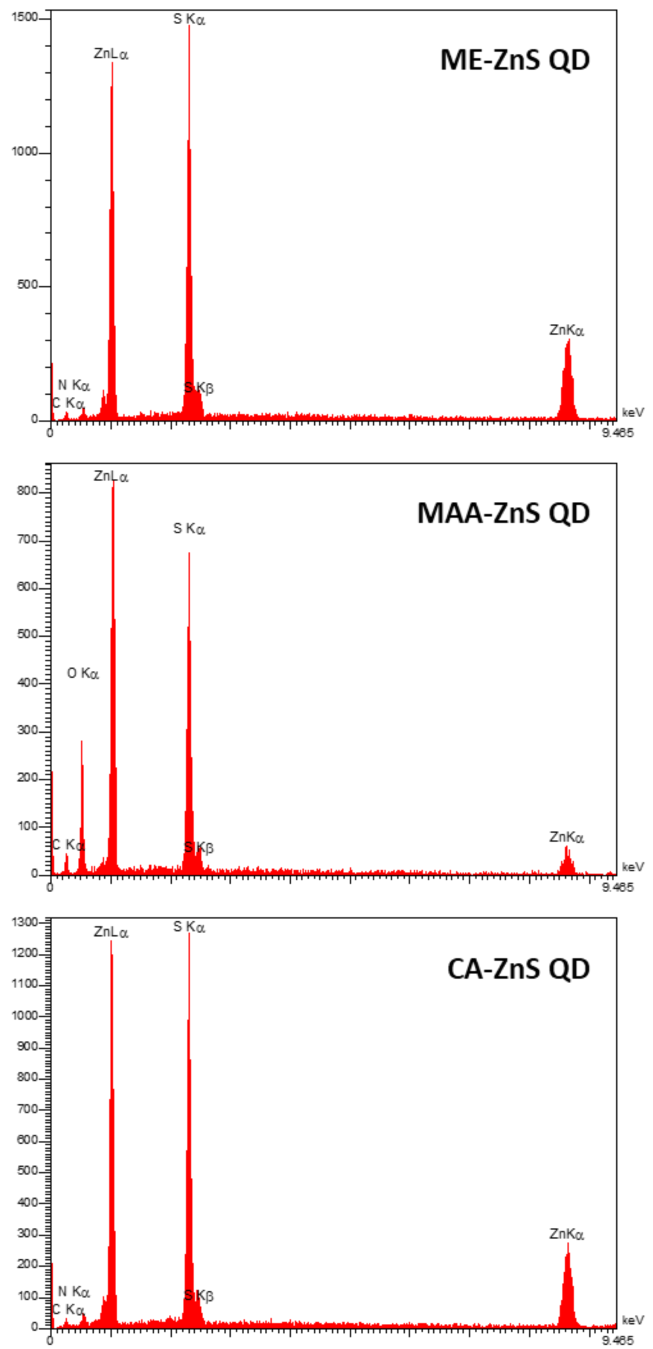

Figure S1. EDX pattern for differently coated ZnS QDs

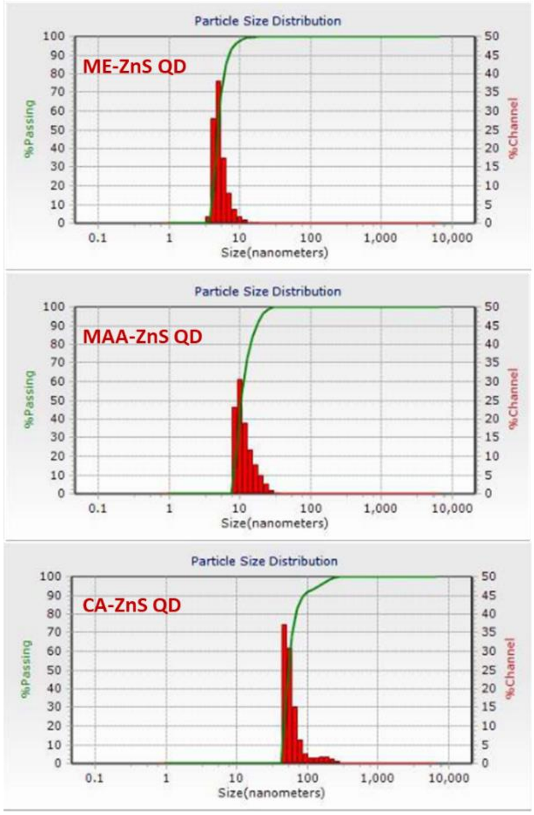

Figure S2 Histogram of hydrodynamic size distribution of ZnS QDs

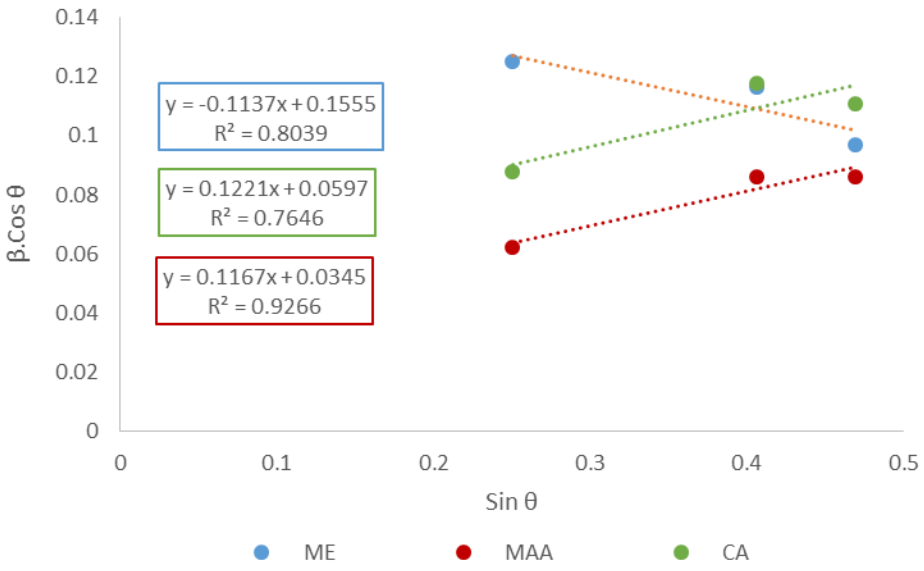

Figure S1. Williamson-Hall graph showing size (intercept) and strain (slope)

Table S1. Measured (Zn) and calculated (S and capping agents) content of coated ZnS quantum dots determined by ICP

Sample	Total conc.	Zn (65.38 g/mol)		S (32.06 g/mol)		Capping agent		Cap:Zn	pk_a-pH	thiol: thiolat
	mg/L	mg/L	µM	mg/L	µM	mg/L	µM			
**ME (78.13 g/mol)**	10	2.79	43	1.38	43	5.83	75	1.7	-2.28	0.005
**MAA (92.11 g/mol)**	10	1.21	18	0.58	18	8.21	89	4.9	-1.5	0.032
**CA (113.61 g/mol)**	10	0.47	7	0.22	7	9.31	82	11.7	1.19	15.488

Table S2. The mode of grain size and sample roughness measured by Atomic Force Microscopy.

Sample	Mode of grain size (nm)	Arithmetic roughness (Ra, nm)	RMS roughness (Rq, nm)	Rz (nm)
**ME-QDs**	28	0.8	5.4	10.4
**MAA-QDs**	35	0.3	2.6	5.8
**CA-QDs**	44	0.1	0.8	1.5

Table S3. The space between different sheets (d) calculated for MAA QDs

Miller indices			Bragg's angle	d-spacing
**h**	k	l	2θ	d=λ/2sinθ
**1**	1	1	29	3.075325455
**2**	2	0	48	1.893116868
**3**	1	1	56	1.640141941
